# Workplace Social Capital and Mental Health among Chinese Employees: A Multi-Level, Cross-Sectional Study

**DOI:** 10.1371/journal.pone.0085005

**Published:** 2014-01-03

**Authors:** Junling Gao, Scott R. Weaver, Junming Dai, Yingnan Jia, Xingdi Liu, Kezhi Jin, Hua Fu

**Affiliations:** 1 School of Public Health, Fudan University, Key Laboratory of Public Health Safety, Ministry of Education, Shanghai, China; 2 School of Public Health, Georgia State University, Atlanta, Georgia, United States of America; Old Dominion University, United States of America

## Abstract

**Background:**

Whereas the majority of previous research on social capital and health has been on residential neighborhoods and communities, the evidence remains sparse on workplace social capital. To address this gap in the literature, we examined the association between workplace social capital and health status among Chinese employees in a large, multi-level, cross-sectional study.

**Methods:**

By employing a two-stage stratified random sampling procedure, 2,796 employees were identified from 35 workplaces in Shanghai during March to November 2012. Workplace social capital was assessed using a validated and psychometrically tested eight-item measure, and the Chinese language version of the WHO-Five Well-Being Index (WHO-5) was used to assess mental health. Control variables included sex, age, marital status, education level, occupation status, smoking status, physical activity, and job stress. Multilevel logistic regression analysis was conducted to explore whether individual- and workplace-level social capital was associated with mental health status.

**Results:**

In total, 34.9% of workers reported poor mental health (WHO-5<13). After controlling for individual-level socio-demographic and lifestyle variables, compared to workers with the highest quartile of personal social capital, workers with the third, second, and lowest quartiles exhibited 1.39 to 3.54 times greater odds of poor mental health, 1.39 (95% CI: 1.10–1.75), 1.85 (95% CI: 1.38–2.46) and 3.54 (95% CI: 2.73–4.59), respectively. Corresponding odds ratios for workplace-level social capital were 0.95 (95% CI: 0.61–1.49), 1.14 (95% CI: 0.72–1.81) and 1.63 (95% CI: 1.05–2.53) for the third, second, and lowest quartiles, respectively.

**Conclusions:**

Higher workplace social capital is associated with lower odds of poor mental health among Chinese employees. Promoting social capital at the workplace may contribute to enhancing employees’ mental health in China.

## Introduction

Social capital is defined as those features of social organization, such as levels of interpersonal trust and the norms of mutual aid and reciprocity, which act as resources for individuals and facilitate collective action [1]–[3]. Although, there remains controversy whether the benefits of social capital accrue to individuals or groups [4], ecological studies have found associations between social capital and health. However, it has been difficult to distinguish between compositional (i.e. individual) and contextual (i.e. group) effects of social capital on health [5]. Thus, it has been suggested that the preferred unit of analysis for conceptualizing and measuring social capital is both the individual and the group [6], [7]. Social capital at the group level most often has been measured by aggregating individual perceptions of social capital [8], [9].

The World Health Organization has defined mental health “as a state of well-being in which every individual realizes his or her own potential, can cope with the normal stressors of life, can work productively and fruitfully, and is able to make a contribution to his or her community” [10]. Mental health is complexly determined by multiple, interacting sociocultural, environmental, psychological and biological factors [10]. Some previous community studies have found empirical support for a positive relationship between social capital and mental health, but this association has not been consistently found across studies [11], [12]. For example, group social capital was associated with mental health in Japan [9] but not in a similar study conducted in the USA [13].

Because many people spend more waking hours at the workplace than elsewhere, and the workplace is a significant source of social relations, it stands to reason that the workplace environment might more appropriately capture the important social interactions and networks that constitute the core of social capital [14]. Whereas previous studies on social capital were conducted in residential or defined geographical areas, it has now been suggested that social capital at work should also be targeted [15], [16]. A multilevel framework is particularly well-suited for studying workplace social capital as it allows for simultaneous examination and disentanglement of effects of workplace-level social capital and of the perceptions of individuals nested within workplaces, thus offering a flexible and comprehensive framework for understanding contextual and compositional effects of social capital on health. However, we are aware of only a few multilevel studies focused on social capital in the workplace [8], [17]. One such study of Finnish employees found that less individual-level social capital was associated with self-reported, physician-diagnosed depression, whereas no contextual effect of workplace-level (aggregated) social capital and depression was observed [18]. Since the meaning of workplace social capital may be culturally bounded [17], [19], further studies are needed to examine the relationships based on employees in different workplaces (e.g., private sectors) and in different cultural settings.

To the best of our knowledge, no published studies on workplace social capital have been conducted in China. The present study attempts to fill this void by using a multilevel framework to examine the association between workplace social capital (both at individual- and aggregated-levels) and mental health among Chinese employees. Generalizing from the extant literature on social capital and mental health conducted in other cultural contexts, we hypothesize that both workplace social capital and individual perceptions of social capital would be associated with better mental health.

## Methods

### Population

The study was conducted in Shanghai, China during March to November 2012. Participants were 2,979 employees from 35 workplaces who were selected using a two-stage sampling procedure. First, we selected 11 governmental agencies, 11 manufacturing worksites, and 13 service companies using a convenience-sampling method. In the second stage, we randomly sampled 100 employees from each workplace that has more than 100 employees; otherwise, in workplaces with fewer than 100 employees, all employees at the workplace were selected. A self-administered questionnaire was distributed by the Human Resources department to all selected employees, whom completed the questionnaire anonymously. The study was approved by the Institutional Review Board of the School of Public Health at Fudan University.

Questionnaires were returned from all selected 35 workplaces and most selected employees. Of the 3,385 employees who were administered the survey, 2,979 (88.0%) returned a completed survey. We excluded from analyses respondents with missing values on the social capital questions, smoking status, sex, or age, which resulted in 2,796 subjects (93.8% of those who returned a completed survey) available for the present study.

### Measurements

#### 1) Mental health

The Chinese language version of WHO-Five Well-Being Index (WHO-5) was used to assess mental health [20]. The WHO-5 has demonstrated excellent psychometric properties in a large representative sample and is widely used and recommended for screening for depression in primary care settings [21].

The WHO-5 consists of five positively worded items that reflect the presence or absence of well-being rather than depressive symptomatology: (1) *I have felt cheerful and in good spirits*, (2) *I have felt calm and relaxed*, (3) *I have felt active and vigorous*, (4) *I woke up feeling fresh and rested*, and (5) *My daily life has been filled with things that interest me*. Participants are asked to report the presence of these positive feelings in the last 2 weeks on a 6-point scale ranging from *all of the time* (5 points) to *at no time* (0 points). A summed score below 13 indicates poor mental health and is an indication for depression [20].

#### 2) Workplace social capital

Workplace social capital was assessed with a validated and psychometrically reliable eight-item measure [22]. Based on the original scale [19], an initial translation into Chinese was done. Then the Chinese translation was back-translated into English to verify that the meaning of the original scale was maintained. Prior psychometric evaluation in Chinese employees has demonstrated the scale to have high internal consistency (Cronbach’s alpha = 0.94) [22]. Using a 5-point Likert scale from 1 =  *Strongly Disagree* to 5 =  *Strongly Agree*, the participants assessed their perceived workplace social capital, defined as the shared values, attitudes, and norms of trust and reciprocity as well as practices of collective action in their workplace [19]. The items were as follows: (1) *We have a “we are together” attitude*; (2) *People feel understood and accepted by each other*; (3) *We can trust our supervisor*; (4) *Our supervisor treats us with kindness and consideration*; (5) *Our supervisor shows concern for our rights as an employee*; (6) *People keep each other informed about work-related issues in the workplace*; (7) *Do members of the workplace build on each other’s ideas in order to achieve the best possible outcome?*; and (8) *People in the workplace cooperate in order to help develop and apply new ideas*.

We assessed the perceived social capital of each employee by calculating the mean score of each individual’s own assessments across the 8 items. Workplace social capital for employee *i* was then measured as the mean perceived social capital for all participating employees from the same workplace as employee *i*, excluding employee *i*. Similar to a previous study [18], both individual- and workplace-level social capital scores were converted into quartiles for the analysis, with the highest quartile indicating the highest level of workplace social capital.

#### 3) Covariates

We selected the following demographic variables as relevant confounders for statistical control: sex, age (10-year categories), marital status (married or cohabiting vs. other), occupational status (public servant vs. other), and education (less than senior high school education vs. more advanced educational attainment). Smoking status (never/former vs. current) and job stress (*Generally speaking, how do you feel your job stress,* with responses ranging from 0 = *low* to 10 = *high*) were also included as covariates. Job stress scores were converted into quartiles for the analyses, with the top quartiles indicating higher levels of job stress. Physical activity was assessed with the Chinese version of the International Physical Activity Questionnaire-short form (IPAQ) [23] and was calculated using the IPAQ analysis algorithms and recommended cutoffs [24]. For this analysis, the physical activity variable was dichotomized into high/moderate versus low/inactive.

### Statistical Analyses

Our data had a multilevel structure comprised of employees (at first level) nested within workplaces (at second level). We fitted the data using multilevel logistic regression models, adjusting for both individual- and workplace-level variables as fixed effects and allowing for a random intercept for mental health. Adjusted odds ratios (ORs) and their 95% confidence intervals (CIs) for poor mental health were obtained for effects of both individual-level and aggregate-level quartiles of workplace social capital. The analyses to examine the association between workplace social capital and mental health involved estimating multiple sequential models [25]. Initially, an unconditional random-intercept model (i.e., empty model without explanatory predictors) was examined to determine whether there was any workplace variation in mental health status. From this model, we computed the proportion of variance in mental health status attributable to the random effect of workplace or the intra-class correlation coefficient (ICC) [25]. The ICC was computed as:
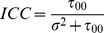
(1)Where 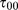 is the workplace level variance (i.e., between-group variance) and 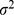 is the individual level variance (within-group variance) for the response variable. In the logistic regression model the level-1 variance is fixed by assumption, 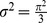.

Next, we estimated a model that included individual-level workplace social capital and the other individual-level covariates (model 1). Then, we estimated a third model that was identical to model except that it substituted workplace-level (aggregated) social capital variable for individual-level social capital (model 2). Finally, we estimated a fourth model that simultaneously included both individual- and workplace-level (aggregated) social capital variables along with all individual level covariates (model 3). We used -2 log likelihood (-2LL) and Akaike information criterion (AIC) to compare the goodness-of-fit of each model [25].

The SAS version 9.1.3 program package was used for all analyses (SAS Institute, Inc., Cary, NC, USA). The multilevel analyses were performed using the GLIMMIX procedure.

## Results

### Descriptive Results

The demographic characteristics and the proportion of participants with poor mental health are shown in Table 1. Of the total sample, 977 workers (34.9%) reported poor mental health. Men, married or cohabiting participants, and workers with low educational level reported poor mental health less frequently than their respective counterparts (*p*<0.005). There were different distributions of poor mental health among different age groups and among participants with different job stress levels (*p*<0.001): Younger workers (<40 years) and those with higher levels of job stress reported higher rates of poor mental health. Mental health also significantly varied among participants with different individual-level social capital (*p*<0.001): Rates of poor mental health declined in conjunction with greater individual perceptions of social capital.

**Table 1:** Descriptive statistics for study variables.

	N(%)	Poor mentalhealth n(%)	*p* value
**All**	2796(100)	977(34.9)	
**Sex**			
Men	1603(57.3)	525(32.8)	.005
Women	1193(42.7)	452(37.9)	
**Education level**			
Low	490(17.5)	121(24.7)	<.001
High	2306(82.5)	856(30.1)	
**Marital status**			
Married or cohabiting	2319(82.9)	783 (33.8)	.004
Other	477(17.1)	194(40.7)	
**Occupational status**			
Public servant	764(27.3)	276(36.1)	.421
Other	2032(72.7)	701(34.5)	
**Smoking status**			
Never/former	1897(67.9)	665(35.1)	.856
Current	899(32.2)	312(34.7)	
**Physical activity**			
High/moderate	2312(82.7)	795(34.4)	.177
Low/inactive	484(17.3)	182(37.6)	
**Age (years)**			
≤29	630(22.5)	274(43.5)	<.001
30–39	774(27.7)	334(43.2)	
40–49	707(25.3)	198(28.0)	
≥50	685(24.5)	171(25.0)	
**Job stress**			
1^st^ quartile (low)	648(23.2)	143(22.1)	<.001
2*nd* quartile	534(19.1)	229(42.9)	
3^rd^ quartile	677(24.2)	245(36.2)	
4^th^ quartile (high)	937(33.5)	360(38.4)	
**Individual-level social capital**			
1^st^ quartile (low)	697(24.9)	360(51.7)	<.001
2nd quartile	408(14.6)	150(36.8)	
3^rd^ quartile	935(33.4)	279(29.8)	
4^th^ quartile (high)	756(27.0)	188(24.9)	

### Multilevel Analyses of the Relationship between Social Capital and Poor Mental Health

The multilevel modeling results are shown in Table 2. The empty model indicated that there was statistically significant variation in mental health status across workplaces (χ^2^(1) = 103.78, *p*<0.001). The interclass correlation coefficient (ICC) was 0.141, indicating that 14.1% of variation in poor mental health was explained by a random effect for workplaces.

**Table 2:** Model fit, odds ratios, and 95% confidence intervals for multilevel regression models of workplace social capital and mental health.

	Empty model	Model 1	Model 2	Model 3
	OR(95% CI)	OR(95% CI)	OR(95% CI)	OR(95% CI)
**Fixed effects**				
**Employee level variables**				
Male		0.80(0.65–1.00)	0.79(0.64–0.98)*	0.80(0.64–0.99)*
Low education level		0.72(0.55–0.94)*	0.70(0.54–0.91)*	0.73(0.56–0.95)*
Other (vs. Married or cohabiting)		1.04(0.80–1.36)	1.04(0.80–1.35)	1.04(0.80–1.36)
Public servant (vs. Other)		0.89(0.70–1.13)	0.86(0.68–1.08)	0.91(0.72–1.15)
Current (vs. never/former) smoking		1.49(1.19–1.87)*	1.50(1.20–1.88)*	1.50(1.19–1.88)*
Active (vs. inactive) physical activity		0.83(0.65–1.07)	0.86(0.67–1.09)	0.85(0.67–1.09)
Age (years)				
≤29		2.39(1.75–3.28)*	2.17(1.60–2.94)*	2.39(1.74–3.27)*
30–39		2.13(1.64–2.77)*	2.06(1.60–2.66)*	2.14(1.65–2.78)*
40–49		1.06(0.81–1.37)	1.06(0.82–1.37)	1.05(0.81–1.37)
≥50		1	1	1
Job stress				
1^st^ quartile (low)		1	1	1
2nd quartile		2.22(1.69–2.92)*	2.43(1.86–3.18)*	2.24(1.71–2.95)*
3^rd^ quartile		2.27(1.64–2.93)*	2.58(1.94–3.44)*	2.28(1.70–3.06)*
4^th^ quartile (high)		2.75(2.06–3.67)*	3.07(2.31–4.08)*	2.87(2.14–3.84)*
Individual-level social capital				
4^th^ quartile (high)		1		1
3^rd^ quartile		1.41(1.12–1.78)*		1.39(1.10–1.75)*
2^nd^ quartile		1.90(1.43–2.54)*		1.85(1.38–2.46)*
1^st^ quartile (low)		3.68(1.24–4.38)*		3.54(2.73–4.59)*
**Workplace-level variable**				
Workplace level social capital				
4^th^ quartile (high)			1	1
3^rd^ quartile			1.13(0.75–1.73)	0.95(0.61–1.49)
2^nd^ quartile			2.33(1.55–3.52)*	1.63(1.05–2.53)*
1^st^ quartile (low)			1.94(1.27–2.96)*	1.14(0.72–1.81)
**Random effects**				
Workplace-level variance (SE)	0.541(0.082)	0.425(0.075)	0.342(0.069)	0.367(0.071)
**ICC**	0.182	0.134	0.036	0.034
**Model fit**				
−2LL	3517.74	3251.98	3349.47	3245.43
AIC	3518.75	3285.98	3383.47	3285.54

*Note*. ICC = interclass correlation coefficient; −2LL = −2 Log Likelihood (smaller is better); AIC = Akaike information criterion (smaller is better).

*p*<.05.

The results of model 1 indicated that the adjusted odds of poor mental health was greater among current smokers, those under 40 years of age, and those with higher education. In addition, there was a positive association between job stress and poor mental health, such that workers in the top 3 quartiles of job stress reported more than twice the odds of poor mental health compared to those workers in the lowest quartile of job stress. Of focal interest, individual-level, perceived social capital was negatively associated with poor mental health after controlling for all individual-level covariates: Compared to workers in the highest quartile of perceived social capital, workers in the lower three quartiles of perceived social capital exhibited progressively greater odds of poor mental health (OR = 1.41–3.68). However, it is possible that at least some of this effect could be due to between-workplace variation in social capital contained within our measurement of individual-level perceptions of social capital.

Hence, we estimated model 2 to examine whether an aggregated workplace social capital variable was a predictor of mental health status. For this model, a similar pattern of individual covariate effects was obtained, except that an additional significant effect for sex was observed: Males had.79 times lower adjusted odds of reporting poor mental health status than females. Of focal interest, workplace-level social capital was significantly associated with mental health status: conditional on the individual-level covariates, employees of workplaces at the first and second quartiles of workplace social capital had 1.94 and 2.33 times greater odds, respectively, of reporting poor mental health than employees of workplaces at the highest quartile of workplace social capital.

In model 3, we added individual-level social capital to model 2. This quasi-contextual model allows us to assess whether individual perceptions of workplace social capital are associated with mental health status after controlling for workplace social capital, and also to assess whether there is a contextual effect of workplace-level social capital (i.e., a differential relationship between social capital and mental health status at the two levels). Results from this model indicated a negative association between individual perceptions of social capital and mental health status with odds ratios similar to those obtained in model 1. At the workplace level, only a small contextual effect for social capital was observed. Employees of workplaces at the 2^nd^ quartile of social capital reported a statistically significant 1.63 times greater odds of poor mental health relative those in workplaces with the highest levels of social capital, after controlling for individual perceptions of social capital and other covariates. There was no significant difference in odds for poor mental health between workplaces with the highest quartile of social capital and those workplaces at the 3^rd^ quartile or lowest quartile of social capital. The pattern of results for the other individual-level covariates was similar to those obtained with model 2. Model 3 exhibited a statistically significant improvement in model fit over model 2 (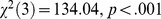) though not in comparison to model 1 (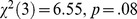). The AIC was lowest for model 3, but it was not appreciably different than model 1. Taken together, the results of these analyses underscore that conclusion that both individual perception and workplace-level measurements of social capital are associated worker reduced odds of poor mental health status, though we found more consistent effects for individual level perceptions of social capital.

## Discussion

To our knowledge, this is the first multilevel modeling study to examine the association between social capital at work and mental health among Chinese employees. Among the other strengths of this study is the use of validated Chinese-language measures of workplace social capital [22] and mental health. The findings suggest that individual-level (perceived) social capital in the workplace is significantly associated with employees’ mental health status after controlling for participants’ socio-demographic characteristics, selected lifestyle variables, and aggregated workplace social capital. When individual-level perceptions of social capital were added to model 2 with aggregated perceptions of workplace social capital, the strength of the relationship of the latter was attenuated and just one contrast (2^nd^ quartile vs. 4^th^ quartile) remained statistically significant (model 3). It indicated that the contextual effect of social capital on mental health might be confounded by the compositional effect of individual perceptions, which was consistent with findings of the study in the USA communities [26]. That is, individual-level perception of social capital may exert a greater influence on mental health than aggregated perceptions do.

Taken together, our results do support the notion that individual perceptions of workplace social capital might protect against poor mental health. These results confirm earlier findings in other culture contexts that individual-level perceptions of social capital may also play a role in shaping workers’ mental health. For example, a previous multilevel prospective study in Finland [18] demonstrated that low individual-level workplace social capital was a predictor of self-reported depression. In addition, a cross-sectional study in Germany [27] also found that low workplace social capital was associated with poor mental health measured by the WHO-5.

The mechanisms underlying the association between individual social capital and mental health in workplace may be largely similar to those in the neighborhood context, including the salience of perceptions [28]. First, high individual social capital at work could buffer the effects of stress by enhancing an individual’s coping abilities [29], [30]. Previous studies have shown workplace social capital was negatively associated with job stress [31], [32], suggesting perhaps that job stress may also mediate the association between social capital and mental health. Secondly, workers in more normative workplaces may find it easier to mobilize various forms of social support from coworkers [33], where support from co-workers could be considered a health resource [34]. Third, more cohesive workplaces are likely to be more effective in maintaining healthy norms and sustaining collective action to reduce workplace health hazards [17]. Conversely, low social capital may be an obstacle for an effective dissemination of mental health information and knowledge at the workplace [33]. Additionally, a low level of integration within a social network may produce negative psychological states, which could decrease motivation for self-care [3], [33], and it could increase vulnerability to the adverse health effects of chronic stress [18].

Our study had several limitations that we should note. First, as is inherent in any cross-sectional study, the ability to draw causal inferences between workplace social capital and employee mental health status is substantially limited. Though we attempted to control for several confounders, we cannot be certain that we have controlled for all possible confounders or determine the directionality of the relationship between social capital and mental health. It is plausible, for instance, that those with depression will tend to perceive their environment, including social capital, more negatively as a result of their depressed mood and associated cognitive distortions. Second, there is a possibility of selection bias caused by non-random sampling (viz., convenience sampling) of workplaces, which may limit the generalizability of the results to other industries. Further longitudinal study investigating the link between workplace social capital and employee mental health status among workers from varied industries is warranted. Third, we did not assess social capital outside the workplace setting. Workplace social capital may be affected by social capital outside workplaces, and vice versa. Indeed, a previous study has shown the importance of considering the social networks at work as well as outside companies on workers’ health [35].

In conclusion, we have found that higher workplace social capital is significantly associated with better mental health among Chinese employees. Prior multilevel studies of the relationship between social capital and health were mainly conducted in Western countries [8]; so, our study advances the existing literature with evidence on the effects of social capital on employee’s mental health from China. Our findings suggest that promoting social capital may contribute to enhancing the employees’ mental health in Chinese workplaces, though further study, particularly longitudinal and intervention research, is needed.
